# A Framework of Covariance Projection on Constraint Manifold for Data Fusion †

**DOI:** 10.3390/s18051610

**Published:** 2018-05-17

**Authors:** Muhammad Abu Bakr, Sukhan Lee

**Affiliations:** Intelligent Systems Research Institute, Sungkyunkwan University, Suwon, Gyeonggi-do 440-746, Korea; abubakr@skku.edu

**Keywords:** Bar-Shalom Campo, Covariance Projection method, data fusion, distributed architecture, Kalman filter, linear constraints, inconsistent data

## Abstract

A general framework of data fusion is presented based on projecting the probability distribution of true states and measurements around the predicted states and actual measurements onto the constraint manifold. The constraint manifold represents the constraints to be satisfied among true states and measurements, which is defined in the extended space with all the redundant sources of data such as state predictions and measurements considered as independent variables. By the general framework, we mean that it is able to fuse any correlated data sources while directly incorporating constraints and identifying inconsistent data without any prior information. The proposed method, referred to here as the Covariance Projection (CP) method, provides an unbiased and optimal solution in the sense of minimum mean square error (MMSE), if the projection is based on the minimum weighted distance on the constraint manifold. The proposed method not only offers a generalization of the conventional formula for handling constraints and data inconsistency, but also provides a new insight into data fusion in terms of a geometric-algebraic point of view. Simulation results are provided to show the effectiveness of the proposed method in handling constraints and data inconsistency.

## 1. Introduction

Data fusion is the process of obtaining a more meaningful and precise estimate of a state by combining data from multiple sources. The architecture of multisensor data fusion can be broadly categorized into two, depending on the way raw data are processed: (1) Centralized fusion architecture [1], where raw data from multiple sources are directly sent to and fused in the central node for state estimation and (2) Distributed fusion architecture [1,2], where data measured at multiple sources are processed independently at individual nodes to obtain local estimates before they are sent to the central node for fusion. In the centralized architecture, it is possible to apply data fusion methodology such as the Kalman filter [3] to all raw data received to yield optimal estimates in the sense of minimum variance. However, the centralized architecture can be costly especially for a large system in terms of infrastructure and communication overheads at the central node, let alone the issues of reliability and scalability. On the other hand, the distributed architecture is advantageous in reliability and scalability, with lower infrastructure and communication costs. Although advantageous, the distributed architecture needs to address statistical dependency among the local state estimates received from multiple nodes for fusion. This is due to the fact that local state estimates at individual nodes can be subject to the same process noise [4] and to double counting, i.e., sharing the same data sources among them [5]. Ignoring such statistical dependency or cross-correlation among multiple nodes leads to inconsistent results, causing divergence in data fusion [6]. In the case of known cross-correlation, the Bar-Shalom Campo (BC) formula [7] provides a consistent fusion result for a pair of redundant data sources, where the fused estimate is based on maximum likelihood [8]. A generalization to more than two data sources with known cross-correlations is given by weighted fusion algorithms of the generalized Millman’s formula (GMF) [9] and weighted Kalman filter (WKF) [10]. 

Sensors often provide spurious and inconsistent data due to unexpected situations such as short duration spike faults, sensor glitches, a permanent failure or slowly developing failure due to sensor elements [5,11]. Since these types of uncertainties are not attributable to the inherent noise, they are difficult to predict and model. The fusion of inconsistent sensor data with correct data can lead to severely inaccurate results [12]. For example, when exposed to abnormalities and outliers, a Kalman filter would easily diverge [13]. Hence, a data validation scheme is required to identify and eliminate the sensor faults/outliers/inconsistencies before fusion. 

The detection of inconsistency needs either a priori information often in the form of specific failure model(s) or data redundancy [5]. Model-based approaches use the generated residuals between the model outputs and actual measurements to detect and remove faults. For instance, in [14], the Nadaraya–Watson estimator and a priori observations are used to validate sensor measurements. Similarly, a priori system model information as a reference is used to detect failures in filtered state estimates [15,16,17]. However, requirement of the prior information restricts the usage of these methods in the general case where prior information is not available or unmodeled failure occurs. A method to detect spurious data based on the Bayesian framework is proposed in [18,19]. The method adds a term to the Bayesian formulation which has the effect of increasing the covariance of the fused probability distribution when measurement from one of the sensor is inconsistent with respect to the other. However, the method is based on heuristics and assumes independence of sensor estimates in its analysis. In [20], the Covariance Union (CU) method is proposed where the fused covariance is enlarged to cover all local means and covariances in such a way that the fused estimate is consistent under spurious data. However, the method incurs high computational cost and results in an inappropriately large conservative fused result. 

In some applications, the state variables observed in a multisensory system may be subject to additional constraints. These constraints can arise due to the basic laws of physics, kinematics or geometry consideration of a system or due to the mathematical relations to satisfy among states. For instance, the energy conservation laws in an undamped mechanical system; Kirchhoff’s laws in electric circuits; a road constraint in a vehicle-tracking scenario [21]; an orthonormal constraint in quaternion-based estimation [22] etc. These constraints if properly included can lead to improvement in state estimation and data fusion. 

Various methods have been proposed to incorporate linear constraints among the state variables of dynamic systems [23,24,25,26,27,28]. For instance, the dimensionality reduction method converts a constrained estimation problem to an unconstrained one of lower dimension by eliminating some state variables using the constraints [25]. However, the state variables in a reduced dimension model may become difficult to interpret and their physical meaning may be lost [23]. The pseudo-measurement method satisfies the linear constraints among state variables by treating the state constraints as additional perfect/noise-free measurements [26,27,28]. However, this method increases the computational complexity of state estimation due to an increase in the dimension of augmented measurement. Furthermore, due to the singularity of augmented measurement covariance, the method may cause numerical problems [23,29]. A popular approach, the estimate projection method, projects the unconstrained estimate obtained from conventional Kalman filtering onto the constraint subspace using classical optimization methods [23,24]. Unfortunately, the method may not lead to the true constraint optimum since the projection method merely gives the solution as a feasible point that is closest to the unconstrained minimum. 

This paper presents a unified and general data fusion framework, referred to as the Covariance Projection (CP) method to fuse multiple data sources under arbitrary correlations and linear constraints as well as data inconsistency. The method projects the probability distribution of true states and measurements around the predicted states and actual measurements onto the constraint manifold representing the constraints to be satisfied among true states and measurements. The proposed method also provides a framework for identifying and removing outliers in a fusion architecture where only sensor estimates may be available at the fusion center. This paper is an extended and improved version of the conference paper [30]. What was presented in [30] is a preliminary new framework of data fusion that we proposed. On the other hand, what is presented here represents a much more detailed implementation and refinement of the concept proposed in the conference paper. Specifically, this paper includes the following additions: (1) a detailed analysis of the equivalence of the proposed method to conventional methods for fusing redundant data sources; (2) handling linear constraints simultaneously under the proposed data fusion framework; (3) refining the mathematical formula and technical descriptions associated with them; and (4) detailed analysis of the method with additional simulations that deal with state estimation and data fusion in the presence of correlations, outliers and constraints.

## 2. Problem Statement

Consider a distributed sensor architecture [1], where each sensor system is equipped with a tracking system to provide local estimates of some quantity of interest in the form of mean and covariance. Assume the following linear dynamic system model for each local sensor system,

(1) $$x_{k}=A_{k-1}\;x_{k-1}+B_{k-1}u_{k-1}+w_{k-1}$$

where k is the discrete time, $A_{k}$ is the system matrix, $B_{k}$ is the input matrix, $u_{k}$ is the input vector and $x_{k}$ is the state vector. The system process is affected by zero mean Gaussian noise $w_{k}$ with covariance matrix Q. The sensor measurements are approximated as,

(2) $$z_{k_{i}}=H_{k_{i}}x_{k}+v_{k_{i}},\;i=1,\ldots ,n$$

where $H_{k}$ is the observation matrix and n represents the number of sensors. $v_{k_{i}}$ is Gaussian noise with covariance matrices $R_{i},\;i=1,2,\ldots ,n$. Each sensor systems employs a Kalman filter to provide local state mean estimate ${\hat{x}}_{k}$ and its covariance $P_{k}$ [31]. A prediction of the state estimate ${\hat{x}}_{k}^{-}$ and its estimation error covariance $P_{k}^{-}$ can be computed based on process model (1),

(3) $${\hat{x}}_{k}^{-}=A_{k-1}\;x_{k-1}+B_{k-1}u_{k-1}$$

(4) $$P_{k}^{-}=A_{k-1}P_{k-1}A_{k-1}^{T}+Q_{k-1}$$

The Kalman filter then provides the state estimate ${\hat{x}}_{k}$ and its covariance $P_{k}$ as,

(5) $${\hat{x}}_{k}={\hat{x}}_{k}^{-}+K_{k}\left(z_{k}-H_{k}{\hat{x}}_{k}^{-}\right)$$

(6) $$P_{k}=\left(I-K_{k}H_{k}\right)P_{k}^{-}{\left(I-K_{k}H_{k}\right)}^{T}+K_{k}R_{k}K_{k}^{T}$$

with the Kalman gain, $K_{k}=P_{k}^{-}H_{k}^{T}{\left(H_{k}P_{k}^{-}H_{k}^{T}+R_{k}\right)}^{-1}$. The estimates provided by sensor systems are assumed to be correlated due to the common process noise or double counting, that is, $P_{ij}=cov\left({\hat{x}}_{i},{\hat{x}}_{j}\right)\neq 0$. To ensure optimality of the fused results, the cross-covariance $P_{ij}$ should be properly incorporated.

Due to the inherent nature of the sensor and environmental factors [32], the sensor measurements can also be perturbed by unmodel random faults $e_{k_{i}}$,

(7) $$z_{k_{i}}=H_{i}x_{k}+v_{k_{i}}+e_{k_{i}},\;i=1,\ldots ,n$$

Subsequently, the estimates computed by local sensor systems may be spurious and inconsistent. Therefore, validation of the sensor estimates is required to remove inconsistencies before the fusion process.

In addition, the states $x_{k}$ of the sensor systems may subject to linear constraints due to the geometry of the system environment or the mathematical description of the system [23], such that,

(8) $$Cx_{k}=c$$

where $C\in {\mathbb{R}}^{n\times m}$ and $c\in {\mathbb{R}}^{n}$ are both known. C is assumed to have a full row rank. The constraints provide deterministic information about the state variables and can be used to improve the fusion accuracy. 

These issues of correlations, data inconsistency and state constraints motivate the development of the Covariance Projection method, which is described next.

## 3. Proposed Approach

**Figure 1:**
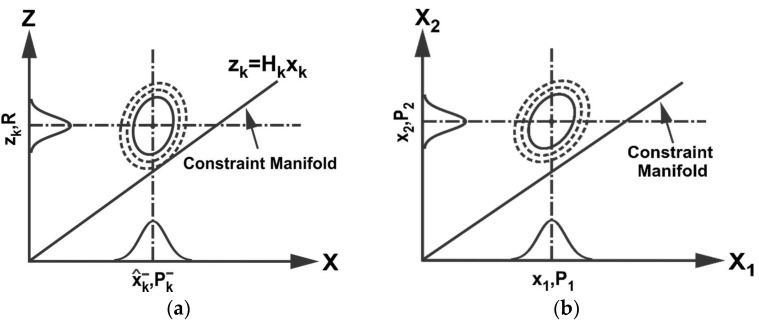
(**a**) Probability of true states and measurements in the extended space around the data from state predictions and sensor measurements and constraint manifold (**b**) Extended space representation of two data sources with constraint manifold.

The proposed method first represents the probability of true states and measurements in the extended space around the data from state predictions and sensor measurements, where the extended space is formed by taking states and measurements as independent variables. Any constraints among true states and measurements that should be satisfied are then represented as a constraint manifold in the extended space. This is shown schematically in Figure 1a for filtering as an example (refer to Equations (1)–(6)). Data fusion is accomplished by projecting the probability distribution of true states and measurements onto the constraint manifold. 

More specifically, consider two mean estimates, ${\hat{x}}_{1}$ and ${\hat{x}}_{2}$, of the state $x\in {\mathbb{R}}^{N}$, with their respective covariances as $P_{1}$, $P_{2}\in {\mathbb{R}}^{N\times N}$. Furthermore, the estimates are assumed to be correlated with cross-covariance $P_{12}$. The mean estimates and their covariances together with their cross-covariance in ${\mathbb{R}}^{N}$ are then transformed to an extended space of ${\mathbb{R}}^{2N}$ along with the linear constraint between the two estimates:

(9) $$\hat{x}=\left[\begin{array}{c} {\hat{x}}_{1}\\ {\hat{x}}_{2} \end{array}\right],P=\left[\begin{array}{cc} P_{1} & P_{12}\\ P_{12}^{T} & P_{2} \end{array}\right],\;C_{1}{\hat{x}}_{1}=C_{2}{\hat{x}}_{2}$$

where $C_{1}$ and $C_{2}$ are constant matrices of compatible dimensions. In the case where ${\hat{x}}_{1}$ and ${\hat{x}}_{2}$ estimate the same entity, $C_{1}$ and $C_{2}$ become identity matrix I. Figure 1b illustrates schematically the fusion of ${\hat{x}}_{1}$ and ${\hat{x}}_{2}$ in the extended space based on the proposed CP method. Fusion takes place by finding the point on the constraint manifold that represents the minimum weighted distance from $\hat{x}$ in ${\mathbb{R}}^{2N}$, where the weight is given by P. As seen later, the proposed CP method with the minimum weighted distance is shown to be equivalent to the minimum variance estimates but advantageous for dealing with additional constraints and data inconsistency.

**Figure 2:**
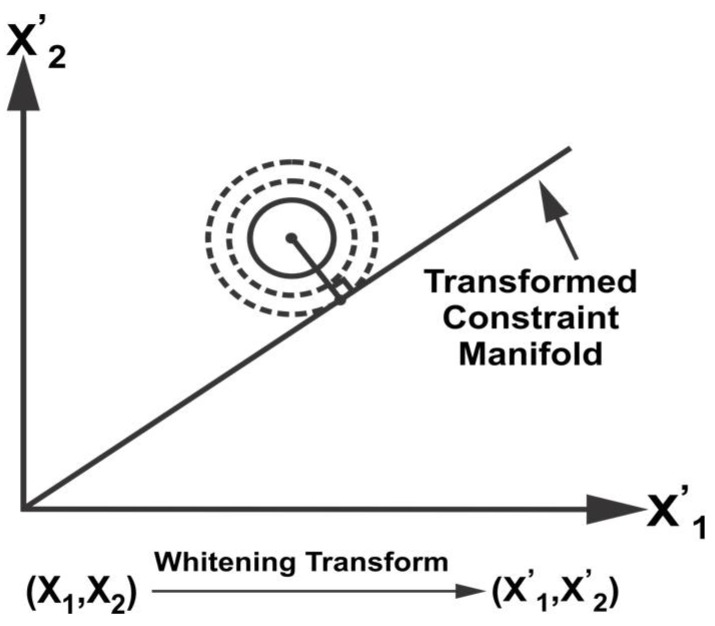
Whitening transform and projection.

To find a point on the constraint manifold with minimum weighted distance, we apply the whitening transform (WT) defined as, $W=D^{-1/2}E^{T}$, where *D* and *E* are the eigenvalue and eigenvector matrices of P. Applying WT,

$${\hat{x}}^{W}=W\hat{x},\;P^{W}=WPW^{T},\;M^{W}=WM$$

where the matrix $M={\left[C_{1}\;C_{2}\right]}^{T}$ is the subspace of the constraint manifold. Figure 2 illustrates the transformation of the probability distribution as an ellipsoid into a unit circle after WT. The probability distribution is then orthogonally projected on the transformed manifold $M^{W}$ to satisfy the constraints between the data sources in the transformed space as illustrated in Figure 2. Inverse WT is applied to obtain the fused mean estimate and covariance in the original space,

(10) $$\tilde{x}=W^{-1}P_{r}W\hat{x}$$

(11) $$\tilde{P}=W^{-1}P_{r}{P_{r}}^{T}W^{-T}$$

where $P_{r}=M^{W}{\left(M^{W^{T}}M^{W}\right)}^{-1}M^{W^{T}}$ is the orthogonal projection matrix. Using the definition of various components in (10) and (11), a close form simplification can be obtained as,

(12) $$\tilde{x}=M{\left(M^{T}P^{-1}M\right)}^{-1}M^{T}P^{-1}\hat{x}$$

(13) $$\tilde{P}=M{\left(M^{T}P^{-1}M\right)}^{-1}M^{T}$$

The details of the simplification are provided in Appendix A. Due to the projection in extended space of ${\mathbb{R}}^{2N}$, (12) and (13) provide a fused result with respect to each data source. In the case where ${\hat{x}}_{1}$ and ${\hat{x}}_{2}$ estimate the same entity, that is, $M={\left[I_{N}\;I_{N}\right]}^{T}$, the fused result will be same for the two data sources. As such, a close form equation for fusing redundant data sources in ${\mathbb{R}}^{N}$ can be obtained from (12) and (13) as,

(14) $$\tilde{x}={\left(M^{T}P^{-1}M\right)}^{-1}M^{T}P^{-1}\hat{x}$$

(15) $$\tilde{P}={\left(M^{T}P^{-1}M\right)}^{-1}$$

Given n mean estimates ${\hat{x}}_{1}$, ${\hat{x}}_{2}$, …, ${\hat{x}}_{n}$ of a state $x\in {\mathbb{R}}^{N}$ with their respective covariances $P_{1},P_{2},\ldots ,P_{n}\in {\mathbb{R}}^{N\times N}$ and known cross-covariances $P_{ij},\;i,j=1,\ldots ,n$, (14) and (15) can be used to obtain the optimal fused mean estimate and covariance with $M={\left[I_{N1},I_{N2},\ldots ,I_{Nn}\right]}^{T}$. 

For fusing correlated estimates from n redundant sources, the CP method is equivalent to the weighted fusion algorithms [9,10], which compute the fused mean estimate and covariance as a summation of weighted individual estimates as,

(16) $$\tilde{x}=\overset{n}{\underset{i=1}{\sum }}c_{i}{\hat{x}}_{i}$$

(17) $$\tilde{P}=\overset{n}{\underset{i,j=1}{\sum }}c_{i}P_{ij}c_{j}^{T}$$

with $\sum_{i=1}^{n}c_{i}=I$. Where the weights $c_{i}$ are determined by solving some cost function of (16) and (17) such that, $\sum_{i=1}^{n}c_{i}=I$. Equivalently, the CP fused mean and covariance can be written as,

(18) $$\tilde{x}=L\hat{x}$$

(19) $$\tilde{P}=LPL^{T}$$

where $L=\left[L_{1},L_{2},\ldots ,L_{n}\right]={\left(M^{T}P^{-1}M\right)}^{-1}M^{T}P^{-1}$ and $\sum_{i=1}^{n}L_{i}=I$. In the particular case of two data sources, the CP fused solution reduces to the well-known Bar-Shalom Campo formula [7],

(20) $$\tilde{x}=\left(P_{2}-P_{21}\right){\left(P_{1}+P_{2}-P_{12}-P_{21}\right)}^{-1}{\hat{x}}_{1}+\left(P_{1}-P_{12}\right){\left(P_{1}+P_{2}-P_{12}-P_{21}\right)}^{-1}{\hat{x}}_{2}$$

(21) $$\tilde{P}=P_{1}-\left(P_{1}-P_{12}\right){\left(P_{1}+P_{2}-P_{12}-P_{21}\right)}^{-1}\left(P_{1}-P_{21}\right)$$

Although equivalent to the traditional approaches in fusing redundant data sources, the proposed method offers a generalized framework not only for fusing correlated data sources but also for handling linear constraints and data inconsistency simultaneously within the framework. 

The proposed method provides an unbiased and optimal fused estimate in the sense of minimum mean square error (MMSE).

**Theorem** **1.** *For* n *unbiased mean estimates* ${\hat{x}}_{1},{\hat{x}}_{2},\ldots ,{\hat{x}}_{n}$ *, the fused mean estimate* $\tilde{x}$ *provided by the CP method is an unbiased estimator of* x *, that is, $E\left(\tilde{x}\right)=E\left(x\right)$.*

**Proof.**  From (18), we can write,

$$\tilde{x}=\left[L_{1},L_{2},\ldots ,L_{n}\right]\left[\begin{array}{c} {\hat{x}}_{1}\\ \begin{array}{c} {\hat{x}}_{2}\\ \vdots \\ {\hat{x}}_{n} \end{array} \end{array}\right]$$

$$\tilde{x}=L_{1}{\hat{x}}_{1}+L_{2}{\hat{x}}_{2}+\ldots +L_{n}{\hat{x}}_{n}$$

$$\tilde{x}=\overset{n}{\underset{i=1}{\sum }}L_{i}{\hat{x}}_{i}$$

Taking the expectation on both sides, we get,

$$E\left(\tilde{x}\right)=E\left(\overset{n}{\underset{i=1}{\sum }}L_{i}{\hat{x}}_{i}\right)$$

$$E\left(\tilde{x}\right)=\overset{n}{\underset{i=1}{\sum }}L_{i}E({\hat{x}}_{i})$$

Since the sensor estimates ${\hat{x}}_{1},{\hat{x}}_{2},\ldots ,{\hat{x}}_{n}$ are unbiased, we have $E({\hat{x}}_{1})=E({\hat{x}}_{2})=\cdots E({\hat{x}}_{n})=E\left(x\right)$,

$$E\left(\tilde{x}\right)=E\left(x\right)$$

where $\sum_{i=1}^{n}L_{i}=I$. This concludes that the fused state estimate $\tilde{x}$ is an unbiased estimate of x. ☐

**Theorem** **2.** *The fused covariance* $\tilde{P}$ *of the CP method is smaller than the individual covariances, that is,* $\tilde{P}\leq P_{i},\;i=1,2,\ldots ,n$ *.*

**Proof.**  From equation (15), we can write,

$$\tilde{P}={\left(M^{T}P^{-1}M\right)}^{-1}$$

By Schwartz matrix inequality, we have,

$$\tilde{P}={\left[{\left(P^{-\frac{1}{2}}M\right)}^{T}\left(P^{\frac{1}{2}}M_{i}\right)\right]}^{T}\times {\left[{\left(P^{-\frac{1}{2}}M\right)}^{T}\left(P^{-\frac{1}{2}}M\right)\right]}^{-1}\times \left[{\left(P^{-\frac{1}{2}}M\right)}^{T}\left(P^{\frac{1}{2}}M_{i}\right)\right]\;\leq {\left(P^{\frac{1}{2}}M_{i}\right)}^{T}\left(P^{\frac{1}{2}}M_{i}\right)=P_{i}$$

where M is the constraint between data sources and $M_{i}={\left[I_{Ni},0,\ldots ,0\right]}^{T}$ is the constraint matrix for $P_{i}$. The equality holds for $P_{i}=P_{ij},$ that is, $\tilde{P}=P_{i},$ when $P_{i}=P_{ij},\;j=1,2,\ldots ,n$.

It can be observed from (14) and (15) that computation of cross-covariance $P_{ij}$ is needed to compute the fused mean and covariance. Cross-covariance among the local estimates can be computed as [9,10,33],

(22) $$P_{ij}=\left[I-K_{i}H_{i}\right]\left[AP_{ij}^{k-1}A^{T}+BQB^{T}\right]{\left[I-K_{j}H_{j}\right]}^{T}$$

where $K_{i}$ and $K_{j}$ are the Kalman gain of source i and j respectively for i,j=1,…,n and $P_{ij}^{k-1}$ represent the cross covariance of the previous cycle between source i and j. ☐

## 4. Fusion in the Presence of Spurious Data

**Figure 3:**
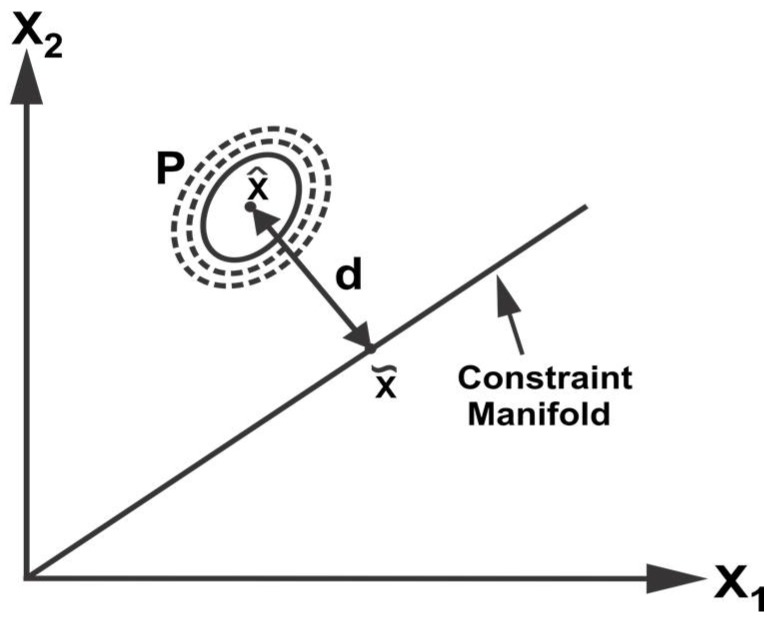
The distance of the multi-variate distribution from the constraint manifold.

Due to the inherent nature of sensor devices and the real-world environment, the sensor observations may also be affected by random faults. Subsequently, the local estimates provided by sensor systems in a distributed architecture may be spurious and inconsistent. This may cause the fusion methodologies to fail since they are based on the assumption of consistent input sensor estimates. Therefore, a validation scheme is required to detect and remove the spurious estimates from the fusion pool. The proposed approach exploits the constraint manifold among sensor estimates to identify any data inconsistency. The identification of inconsistent data is based on the distance from the constraint manifold to the mean of redundant data sources in the extended space that provides a confidence measure with the relative disparity among data sources. Assuming a joint multivariate normal distribution for the data sources, the data confidence can be measured by computing the distance from the constraint manifold as illustrated in Figure 3. 

Consider the joint space representation of n sensor estimates $\left({\hat{x}}_{N1},P_{1}\right),\left({\hat{x}}_{N2},P_{n}\right),\ldots ,\left({\hat{x}}_{Nn},P_{1}\right)$,

$$\hat{x}=\left[\begin{array}{c} {\hat{x}}_{N1}\\ \begin{array}{c} {\hat{x}}_{N2}\\ \vdots \\ {\hat{x}}_{Nn} \end{array} \end{array}\right],P=\left[\begin{array}{cc} P_{1} & \begin{array}{ccc} P_{12} & \ldots & P_{1n} \end{array}\\ \begin{array}{c} P_{12}^{T}\\ \vdots \\ P_{1n}^{T} \end{array} & \begin{array}{ccc} \begin{array}{c} P_{2}\\ \vdots \\ \ldots \end{array} & \begin{array}{c} \ldots \\ \ddots \\ \ldots \end{array} & \begin{array}{c} \vdots \\ \vdots \\ P_{nn} \end{array} \end{array} \end{array}\right]$$

where N is the dimension of the state vector. The distance d from the constraint manifold can be calculated as,

(23) $$d={\left(\hat{x}-\tilde{x}\right)}^{T}P^{-1}\left(\hat{x}-\tilde{x}\right)$$

where $\tilde{x}$ is the point on the manifold and can be obtain by using (12). In the case of two data sources with mean ${\hat{x}}_{1}$, ${\hat{x}}_{2}$ ∈ ${\mathbb{R}}^{N}$ and respective covariance matrices $P_{1}$ and $P_{2}$ ∈ ${\mathbb{R}}^{N\times N}$. The distance d can be obtained as,

$$d=\left[\begin{array}{cc} {\left({\hat{x}}_{1}-\tilde{x}\right)}^{T} & {\left({\hat{x}}_{2}-\tilde{x}\right)}^{T} \end{array}\right]{\left[\begin{array}{cc} P_{1} & 0\\ 0 & P_{2} \end{array}\right]}^{-1}\left[\begin{array}{c} {\hat{x}}_{1}-\tilde{x}\\ {\hat{x}}_{2}-\tilde{x} \end{array}\right]$$

The point on the manifold is given as,

$$\tilde{x}=P_{2}{\left(P_{1}+P_{2}\right)}^{-1}{\hat{x}}_{1}+P_{1}{\left(P_{1}+P_{2}\right)}^{-1}{\hat{x}}_{2}$$

Simplifying, we get,

(24) $$d={\left[{\hat{x}}_{1}-{\hat{x}}_{2}\right]}^{T}{\left(P_{1}+P_{2}\right)}^{-1}\left[{\hat{x}}_{1}-{\hat{x}}_{2}\right]$$

The details of simplifications are provided in Appendix B. From (24), it can be observed that distance d is a weighted distance between the two data sources and it can provide a measure of nearness or farness of the two data sources to each other. A large value of d implies a large separation while a small d signifies closeness of the data sources. In other words, the distance from the manifold provides an indication of the relative disparity among data sources.

**Theorem** **3.** *For* n *data sources of* N *dimension, the* d *distance (23) follow a chi-squared distribution with* nN *degrees of freedom (DOF), that is,* $d\sim \chi^{2}\left(Nn\right)$ *.*

**Proof.**  From (23) we have,

(25) $$d={\left(\hat{x}-\tilde{x}\right)}^{T}P^{-1}\left(\hat{x}-\tilde{x}\right)$$

Under the whitening transformation, $WP^{-1}W=I$, $W\hat{x}={\hat{x}}^{W}$ and $W\tilde{x}={\tilde{x}}^{W}$. Thus, we can write,

(26) $${\left(\hat{x}-\tilde{x}\right)}^{T}P^{-1}\left(\hat{x}-\tilde{x}\right)={\left({\hat{x}}^{W}-{\tilde{x}}^{W}\right)}^{T}\left({\hat{x}}^{W}-{\tilde{x}}^{W}\right)\;\Rightarrow {\left(W\left(\hat{x}-\tilde{x}\right)\right)}^{T}\left(W\left(\hat{x}-\tilde{x}\right)\right)=y^{T}y$$

where for normal distribution $\left(\hat{x}-\tilde{x}\right)$, $y=W\left(\hat{x}-\tilde{x}\right)$ is an independent standard normal distribution N(0,1). For *N* dimensions of the state vector, the right-hand side of (26) is $\sum_{i=1}^{N}y_{i}^{2}$, thus distance d follows a chi-square distribution with N DOF, that is, $d\sim \chi^{2}\left(N\right)$. For n data sources with N states,

$$d\sim \chi^{2}\left(Nn\right)$$

Since d is a chi-square distribution with Nn DOF, then for any significance level α∈(0,1), $\chi_{\alpha }^{2}\left(Nn\right)$ is defined such that the probability,

$$P\left\{d\geq \chi_{\alpha }^{2}\left(Nn\right)\right\}=\alpha$$

**Figure 4:**
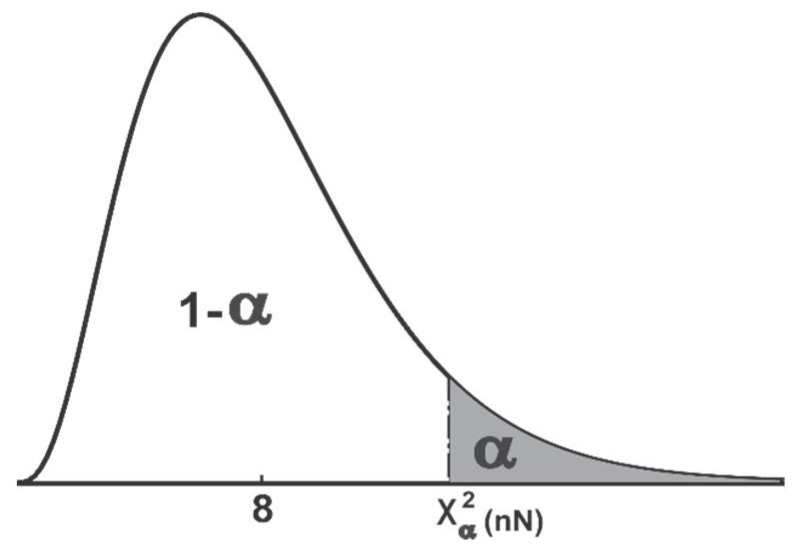
Chi-square distribution with 8 degreed of freedom. The unshaded area represents a cumulative probability associated with chi-square statistics $\chi_{\alpha }^{2}\left(Nn\right)$.

This is depicted in Figure 4. Hence, to have a confidence of 100 × (1 − α) percent, d should be less than respective critical value as illustrated in Figure 4. A chi-square table [34] can be used to obtain the critical value for the confidence distance with a particular significance level and DOF. A value of α = 0.05 is assumed in this paper unless specified. ☐

### 4.1. Inconsistency Detection and Exclusion

To obtain reliable and consistent fusion results, it is important that the inconsistent estimates be identified and excluded before fusion. For this reason, at each time step when the fusion center receives computed estimates from sensor nodes, distance d is calculated. A chi-square table is then used to obtain the critical value for a particular significance level and DOF. A computed distance d less than the critical value mean that we are confident about the closeness of sensor estimates and that they can be fused together to provide a better estimate of the underlying states. On the other hand, a distance d greater than or equal to the critical value indicate spuriousness of the sensor estimates. At least one of the sensor estimate is significantly different than the other sensor estimates. To exclude the outliers, a distance from the manifold is computed for every estimate and compared with the respective critical values. For n mean estimates ${\hat{x}}_{1},{\hat{x}}_{2},\ldots ,{\hat{x}}_{n}$ with respective covariances $P_{1},P_{2},\ldots ,P_{n}$ and cross-covariances $P_{ij}\;\mathrm{for}\;i,j=1,\ldots ,n$, the hypothesis and decision rule are summarized as follows,

**Hypotheses:**  $$H_{0}:\;{\hat{x}}_{1}={\hat{x}}_{2}=\cdots ={\hat{x}}_{n}$$

$$H_{1}:\;{\hat{x}}_{1}\neq {\hat{x}}_{2}\neq \cdots \neq {\hat{x}}_{n}$$

**Compute:**  $d={\left(\hat{x}-\tilde{x}\right)}^{T}P^{-1}\left(\hat{x}-\tilde{x}\right)$

**Decision Rule:**  Accept $H_{0}$ if $d<\chi_{\alpha }^{2}\left(Nn\right)$

       Reject $H_{0}$ if $d\geq \chi_{\alpha }^{2}\left(Nn\right)$

If the hypothesis $H_{0}$ is accepted, then the estimates are optimally fused using (14) and (15). On the other hand, rejection of null hypothesis means that at least one of the sensor estimates is significantly different than the other estimates. Then, a distance from the manifold is computed for each of the estimates as,

$$d_{i}={\left({\hat{x}}_{i}-\tilde{x}\right)}^{T}P_{i}^{-1}\left({\hat{x}}_{i}-\tilde{x}\right),\;i=1,2,\ldots ,n$$

with $\tilde{x}$ computed using (14). The outliers are identified and eliminated based on the respective critical value, that is, if $d_{i}\geq \chi_{\alpha }^{2}\left(N\right)$, they are rejected, where N is the dimension of an individual data source.

### 4.2. Effect of Correlation on d Distance

Since the estimates provided by multiple data sources are correlated, it is important to consider the effect of cross-correlation in the calculation of confidence distance. Consider two sensor estimates ${\hat{x}}_{1}\in {\mathbb{R}}^{1}$ and ${\hat{x}}_{2}\in {\mathbb{R}}^{1}$ with respective variances $\sigma_{1}^{2}$ and $\sigma_{2}^{2}$ and cross-covariance $\sigma_{12}^{2}=\rho \sqrt{\sigma_{1}^{2}\sigma_{2}^{2}}$, where ρ∈[−1,1] is the correlation coefficient. The d distance for the pair of multivariate Gaussian estimates $\left({\hat{x}}_{1},\sigma_{1}^{2}\right)$ and $\left({\hat{x}}_{2},\sigma_{2}^{2}\right)$, with cross-covariance $\sigma_{12}^{2}$ can be written as,

(27) $$d=\frac{{\left[{\hat{x}}_{1}-{\hat{x}}_{2}\right]}^{2}}{\sigma_{1}^{2}+\sigma_{2}^{2}-\sigma_{12}^{2}-\sigma_{21}^{2}}$$

**Figure 5:**
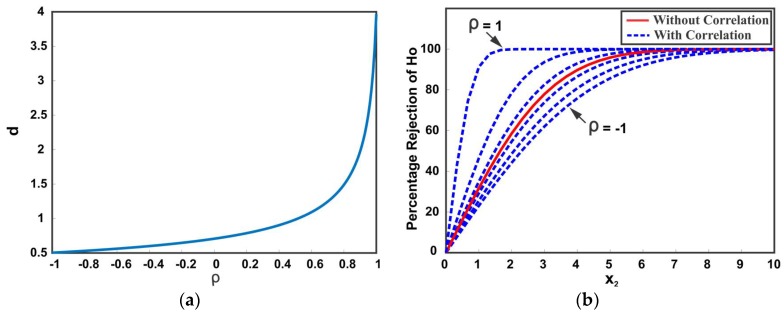
Effect of correlation on d distance (**a**) *d* distance with correlation ρ∈[−1,1]; (**b**) percentage of rejecting the null hypothesis $H_{0}$ with different correlation values.

It is apparent that the distance between the mean values is affected by the correlation between the data sources. Figure 5 illustrates the dependency of confidence distance d on the correlation coefficient. Figure 5a shows the distance d with changing correlation coefficient from −1 to 1. It can be observed that a positive correlation between the data sources results in a large d distance. This means that a slight separation between the positively correlated data sources indicates spuriousness with high significance as compared to negatively correlated and uncorrelated data sources. Figure 5b shows the scenario in which a data source (with changing mean and constant variance) is moving away from another data source (with constant mean and constant variance). The distance d is plotted for various values of correlation coefficients. The y-axis shows the percentage of rejection of the null hypothesis $H_{0}$. It can be noted that ignoring the cross-correlation in distance d results in underestimated or overestimated confidence and may lead to an incorrect rejection of the true null hypothesis (Type I error) or incorrect retaining of false null hypothesis (Type II error). The proposed framework inherently takes care of any cross-correlation among multiple data sources in the computation of distance d.

**Example:**  *Consider a numerical simulation with the constant state,*

$$x_{k}=10$$

Three sensors are used to estimate the state $x_{k}$, where the measurements of the sensors are affected with respective variances of $R_{1},\;R_{2}\;\mathrm{and}\;R_{3}$. The values for the parameters assumed in the simulation are,

$$Q=2,\;R_{1}=0.5,\;R_{2}=1,\;R_{3}=0.9$$

**Figure 6:**
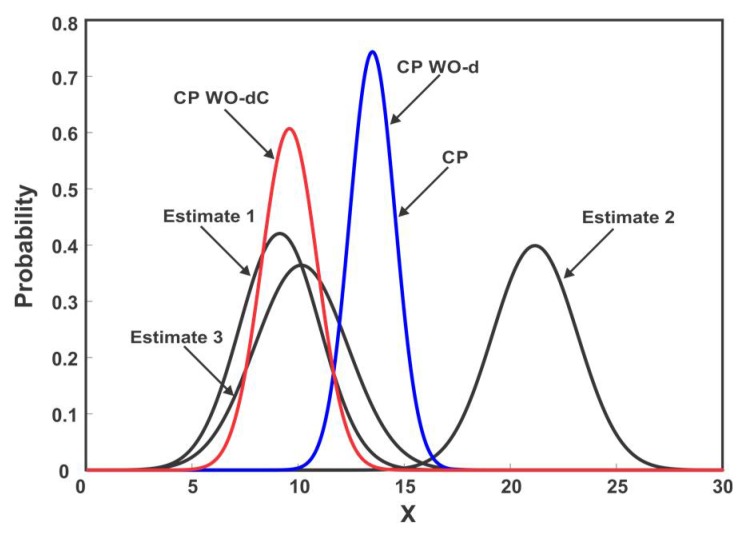
Three-sensor fusion when the estimate of sensor 2 is inconsistent. Neglecting the cross-correlation results in Type II error.

**Figure 7:**
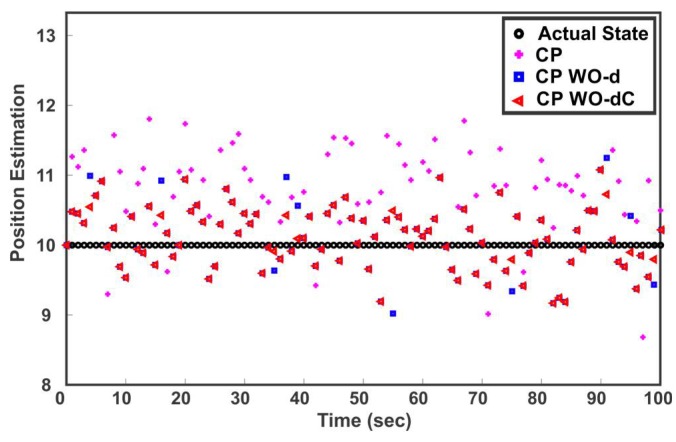
Estimated position after three-sensor fusion in presence of inconsistent estimates.

The sensor measurements are assumed to be cross-correlated. It is also assumed that the sensor 1, sensor 2 and sensor 3 measurements are independently corrupted by unmodeled noise and produce inconsistent data for 33%, 33% and 34% of the time respectively. The sensors compute local estimates of the state and send it to the fusion center. Three strategies for combining the local sensor estimates are compared: (1) CP, which fuses the three sensor estimates using (14) and (15) without removing outliers; (2) CP WO-d means the outliers were identified and rejected before fusion based on (27) with $\sigma_{12}^{2}=0$, that is, correlation in computation of d is ignored and; (3) CP WO-dC, reject the outliers based on (27) with taking into account the cross-correlation. Figure 6 shows the fused solution of three sensors when the estimate provided by sensor 2 is in disagreement with sensor 1 and 3. It can be observed from Figure 6 that neglecting the cross-correlation in CP WO-d results in Type II error, that is, all the three estimates are fused despite the fact that estimate 2 is inconsistent. CP WO-dC correctly identifies and eliminates the spurious estimate before the fusion process. Figure 7 shows the estimated position after the fusion of the three sensors’ estimates for 100 samples. It can be seen that the presence of outliers greatly affects the outcome of multisensor data fusion. As depicted in Figure 7, eliminating outliers before fusion improves the estimation performance. The fused samples of CP WO-d and CP WO-dC on average lies closer to the actual state. Figure 7 also shows the difference in fusion performance when outliers are identified with and without considering cross-correlation. It can be noted that neglecting the correlation affects the estimation quality because of Type I and Type II errors.

## 5. Fusion under Linear Constraints

The system model of a linear dynamic system takes into account the relation and dependencies among components of the state vector. In some applications, however, the state variables may be subject to additional constraints due to the basic laws of physics, geometry of the system environment or due to the mathematical description of the state vector. Imposing such certain information in an otherwise probabilistic setting should yield a more accurate estimate that is guaranteed to be feasible. 

Consider a linear dynamic system model,

(28) $$x_{k}=A_{k-1}\;x_{k-1}+B_{k-1}u_{k-1}+w_{k-1}$$

(29) $$z_{k_{i}}=H_{k_{i}}x_{k}+v_{k_{i}},\;i=1,\ldots ,n$$

where k represents the discrete-time index, $A_{k}$ is the system matrix, $B_{k}$ is the input matrix, $u_{k}$ is the input vector and $x_{k}$ is the state vector. The system process noise $w_{k}$ with covariance matrix Q and measurement noise $v_{k}$ with covariance R are assumed to be correlated, that is,

$$E\left\{\left[\begin{array}{c} w_{k}\\ v_{k} \end{array}\right]{\left[\begin{array}{c} w_{k}\\ v_{k} \end{array}\right]}^{T}\right\}=\left[\begin{array}{cc} Q & P_{QR}\\ P_{QR}^{T} & R \end{array}\right]$$

The state $x_{k}\in {\mathbb{R}}^{N}$ is known to be constrained as,

(30) $$Cx_{k}=c=0$$

For c≠0, the state space can be translated by a factor c such that $C{\bar{x}}_{k}=0$. After constrained state estimation, the state space can be translated back by the factor c to satisfy $Cx_{k}=c$. Hence, without loss of generality, the c=0 case is considered for analysis here. The matrix $C\in {\mathbb{R}}^{n\times m}$ is assumed to have a full row rank. A row deficient matrix C signifies the presence of redundant constraints. In such a case, we can simply remove the linearly dependent rows from C. In the following, the estimate projection (EP) method is briefly reviewed which is followed by the Covariance Projection (CP) method for linear constraints among state variables.

### 5.1. Estimate Projection Method

The estimate projection (EP) method [23,24] projects the unconstrained estimate obtained from Kalman filtering onto the constraint subspace to satisfy the linear constraints among state variables. Let us denote the unconstrained filtered estimate and constrained estimate as $\left({\hat{x}}^{u},P^{u}\right)$ and $\left({\hat{x}}^{p},P^{p}\right)$, respectively. Then the following optimization problem is solved to obtain the constrained estimate,

(31) $$\underset{{\hat{x}}^{p}}{\min}{\left({\hat{x}}^{p}-{\hat{x}}^{u}\right)}^{T}U\left({\hat{x}}^{p}-{\hat{x}}^{u}\right)\;\mathrm{such}\;\mathrm{that}\;C{\hat{x}}^{p}=0$$

where U is any symmetric positive definite weighting matrix. Solving (31) using Lagrange multipliers results in a constrained mean and covariance,

(32) $${\hat{x}}^{p}=J{\hat{x}}^{u}$$

(33) $$P^{p}=JP^{u}J^{T}$$

where J is the projector on the null space of constrained matrix C, defined as,

$$J=I-U^{-1}C^{T}{\left(CU^{-1}C^{T}\right)}^{-1}C$$

Any symmetrical positive definite matrix can be used as a weighting matrix U to obtain the constrained estimate but the two most common choices are identity matrix I and inverse of unconstrained covariance $P^{u^{-1}}$.

### 5.2. Covariance Projection Method for Linear Constraints

The CP framework incorporates any linear constraints among states without any additional processing. Let us denote the constrained filtered estimate of the CP method as $\left({\hat{x}}^{c},P^{c}\right)$. The extended space representation of the state predictions and measurements of multiple sensors can be written as,

$$\hat{x}=\left[\begin{array}{c} {\hat{x}}_{k}^{-}\\ z_{k_{1}}\\ \begin{array}{c} \vdots \\ z_{k_{n}} \end{array} \end{array}\right],\;P=\left[\begin{array}{cc} P_{k}^{-} & \begin{array}{ccc} P_{P_{k}^{-}R_{1}} & \ldots & P_{P_{k}^{-}R_{n}} \end{array}\\ \begin{array}{c} P_{P_{k}^{-}R_{1}}^{T}\\ \vdots \\ P_{P_{k}^{-}R_{n}}^{T} \end{array} & \begin{array}{ccc} \begin{array}{c} R_{1}\\ \vdots \\ \ldots \end{array} & \begin{array}{c} \ldots \\ \ddots \\ \ldots \end{array} & \begin{array}{c} \vdots \\ \vdots \\ R_{n} \end{array} \end{array} \end{array}\right]$$

Then the CP estimate in the presence of linear constraints among states can be obtained using (12) and (13) as,

(34) $${\hat{x}}^{c}=M_{c}{\left(M_{c}^{T}P^{-1}M_{c}\right)}^{-1}M_{c}^{T}P^{-1}\hat{x}$$

(35) $$P^{c}=M_{c}{\left(M_{c}^{T}P^{-1}M_{c}\right)}^{-1}M_{c}^{T}$$

where the $M_{c}$ matrix is the subspace of the constraint among the state prediction $x_{k}^{-}$ and sensor measurements $z_{k_{i}}$ as well as linear constraints C among state variables. The subspace of the linear constraint among state prediction and sensor measurements can be written as,

$$M={\left[I_{N},H_{1},H_{2},\ldots ,H_{n}\right]}^{T}$$

Then, $M_{c}$ is a combination of M and C, that is,

$$M_{c}\in \left(M,C\right)$$

The projection of the probability distribution of true states and measurements around the predicted states and actual measurements onto the constraint manifold $M_{c}$ in the extended space provide the filtered or fused estimate of state prediction and sensor measurements as well as completely satisfying the linear constraints among the states directly in one step. 

It can be observed from (31) that the EP method forces the unconstrained estimate on the linear constraint under some norm to satisfy the linear constraints among state variables. Consequently, the true optimality cannot be guaranteed due to the fact that the projected point close to the unconstrained estimate does not imply that it is close to the true constrained state [29]. On the other hand, the unified constraint matrix $M_{c}$ of the CP method ensures that the linear constraints among state variables are exactly satisfied. Furthermore, as compared to the EP method that needs the online projection steps (32) and (33) after filtering, the $M_{c}$ matrix for the CP method can be computed offline. This means that the EP method is computationally less efficient. Additionally, the proposed CP method is inherently suitable for taking care of any cross-correlation in the constrained estimation process.

## 6. Simulation Results

In this section, illustrative examples are provided to demonstrate the effectiveness of the theoretical results derived in the previous sections. We use the Monte Carlo technique [35], a method extensively used in a wide variety of fields such as physical science, computational biology, statistics, computational geometry, artificial intelligence, engineering, decision theory, and quantitative finance (see, e.g., the recent works [36,37,38,39,40]). 

### 6.1. Tracking in the Presence of Correlations and Outliers

Consider a target tracking scenario characterized by the following dynamic system model,

(36) xk=[1T01]xk−1+[T22T]wk−1

with the state vector $x_{k}={\left[x,\dot{x}\right]}^{T}$. Where x and $\dot{x}$ are the position and velocity of the target at time k, respectively. *T* = 0.5 s is the sampling period. The system process is affected by zero mean Gaussian noise with covariance matrix Q. Four sensors are employed to track the movement of the target, where the sensor measurements are approximated by the following equation,

(37) $$z_{k_{i}}=\left[\begin{array}{cc} 1 & 0\\ 0 & 1 \end{array}\right]x_{k}+v_{k_{i}},\;i=1,2,3,4$$

The measurements of the sensors are affected by noise $v_{k_{i}}$ with respective covariances of $R_{1},\;R_{2},\;R_{3}\;\mathrm{and}\;R_{4}$. The covariances of the process noise and sensor measurement noises used in the simulation are,

$$Q=3.5,\;R_{1}=diag\left(5,3.5\right),\;R_{2}=diag\left(2,8\right),$$

$$R_{3}=diag\left(7,2.1\right),\;R_{4}=diag\left(2.5,5\right)$$

Starting from an initial value of [100, 3], in each time step the individual sensor uses (36) to predict the state of the target and then update the state prediction by its own sensor measurements. The local estimates are assumed to be correlated and (22) is used to compute the cross-correlation among local estimates. The estimated mean and covariances of the states by each sensor are sent to the fusion center, where they are fused by the CP method. Table 1 summarizes the trace of covariance of local sensors along with the trace of fused covariance provided by the CP method. As seen from Table 1, the trace of covariance provided by the CP method is less than the individual local state estimates, that is, $trace\tilde{P}\leq$ $traceP_{i}$, i=1,…,4. This means that the fused result is better than each of the local state estimates. 

In order to further verify this theoretical result, we compute the mean square error (MSE) as,

$$S_{MSE}\left(k\right)=\frac{1}{V}\overset{V}{\underset{i=1}{\sum }}{\left[{\hat{x}}_{i}\left(k\right)-x_{i}\left(k\right)\right]}^{T}\left[{\hat{x}}_{i}\left(k\right)-x_{i}\left(k\right)\right]$$

where *V* is the number of Monte Carlo trials and ${\hat{x}}_{i}\left(k\right)$ and $x_{i}\left(k\right)$ are the estimated and actual state vector respectively. Since,

$$trace\left(P_{i}\right)=E\left[{\left({\hat{x}}_{i}-x\right)}^{T}\left({\hat{x}}_{i}-x\right)\right]$$

Then we have [41],

(38) $$S_{MSE}\left(k\right)=trace\left(P_{i}\right),\;k\to \infty ,\;V\to \infty$$

**Figure 8:**
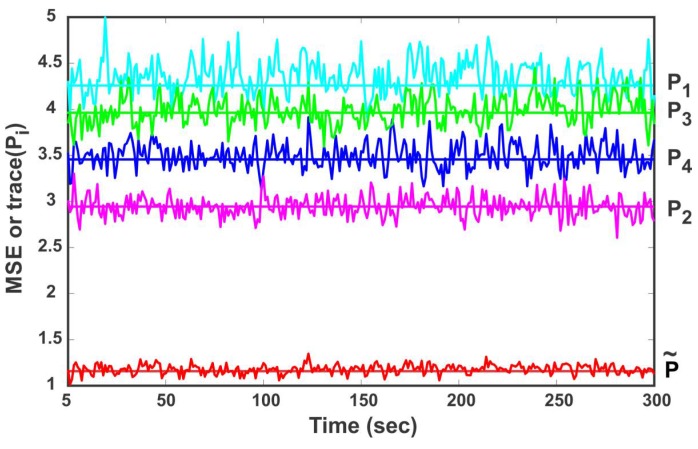
The mean square error (MSE) and trace ($P_{i}$) of local and fused estimates.

The simulation is carried out for 1000 Monte Carlo runs and the local estimates provided by four sensor nodes along with the fused result of the CP method are shown in Figure 8. The straight lines in Figure 8 denote the trace of error covariance matrices and the solid curve represents the MSE of local and fused estimates. It can be observed from Figure 8 that the MSE of the individual sensor node fluctuates around the trace, which is consistent with (38). Furthermore, the accuracy relation of local sensor estimates and fused estimates in terms of MSE in Figure 8 is coincident with the theoretical results in Table 1.

**Figure 9:**
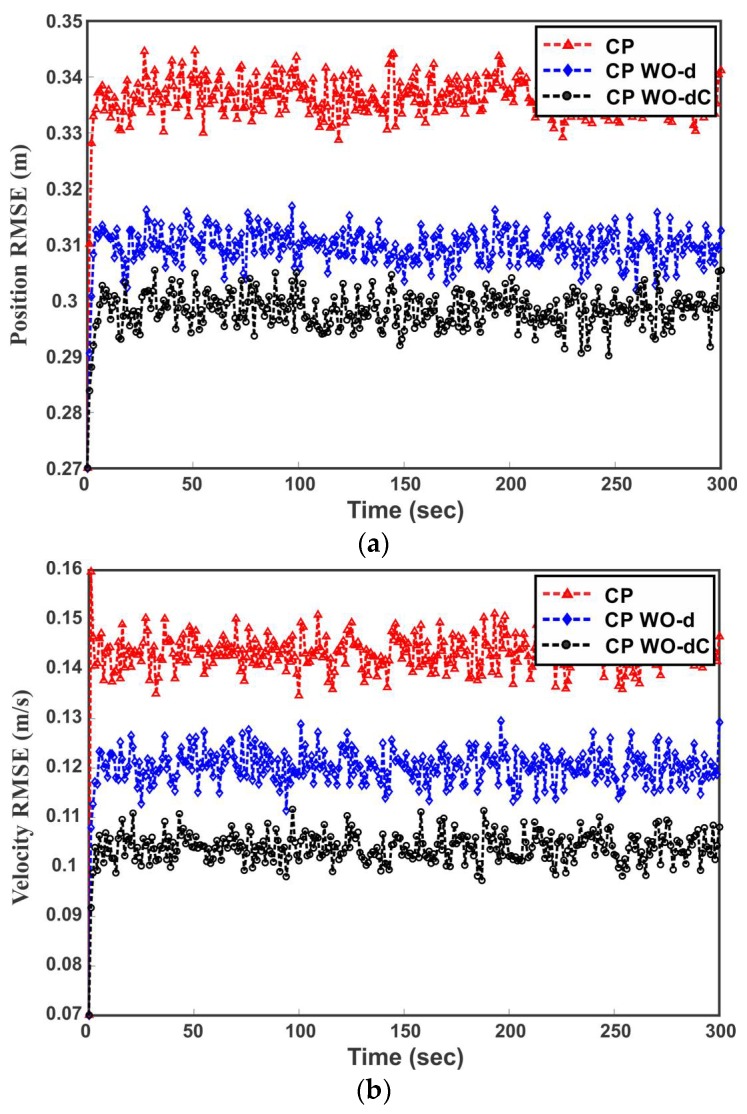
Illustration of multisensor data fusion in the presence of inconsistent estimates. (**a**) Position root mean squared error (RMSE); (**b**) velocity RMSE.

Consider the same dynamic example of four sensors with the following system and measurement equations,

(39) xk=[1T01]xk−1+[T22T]uk−1+wk−1

(40) $$z_{k_{i}}=\left[\begin{array}{cc} 1 & 0\\ 0 & 1 \end{array}\right]x_{k}+v_{k_{i}}+e_{k_{i}},\;i=1,2,3,4$$

where the process and measurement noise parameters are the same. Now, it is also assumed that the sensor 1, sensor 2, sensor 3 and sensor 4 measurements are independently affected by unmodeled random noise $e_{k_{i}}$ for 5%, 15%, 20% and 10% of the time, respectively, and thus the estimates provided by the sensors are spurious. The control input alternate between 1 and −1 and set to a value of 1 if $\dot{x}<30$ otherwise it is changed to −1 until $\dot{x}<5$. Starting from an initial value of [100, 3], in each time step the individual sensor node compute local filtered estimates. The estimated mean and covariances by each sensor are sent to the fusion center, where they are fused. The three fusion strategies of CP (fusion without outlier removal), CP WO-d (outlier removal without considering cross-correlation) and CP WO-dC (taking care of correlation in outlier removal) are compared based on root mean squared error (RMSE) between the actual state value and fused estimate of the state. The inconsistency is detected with significance level α = 0.05. Figure 9a,b illustrate the RMSE of the target position and velocity respectively versus time for 1000 Monte Carlo runs. Table 2 summarizes the average RMSE of position and velocity. It can be observed from Figure 9 and Table 2 that the presence of outliers deteriorates the performance of multisensor data fusion. Eliminating the outliers before fusion greatly improves the estimation quality. Figure 9 and Table 2 also shows the difference in fusion performance of CP WO-d and CP WO-dC, when outliers are identified with and without consideration of cross-correlation in distance d respectively. It can be noted that consideration of correlation in distance d improves the estimation quality in presence of outliers by avoiding Type I and Type II errors.

### 6.2. Target Tracking in the Presence of Linear Constraints

Consider a 2D target tracking problem [24] with the following system equation,

(41) $$x_{k}=\left[\begin{array}{ccc} 1 & 0 & \begin{array}{cc} T & 0 \end{array}\\ 0 & 1 & \begin{array}{cc} 0 & T \end{array}\\ \begin{array}{c} 0\\ 0 \end{array} & \begin{array}{c} 0\\ 0 \end{array} & \begin{array}{cc} \begin{array}{c} 1\\ 0 \end{array} & \begin{array}{c} 0\\ 1 \end{array} \end{array} \end{array}\right]x_{k-1}+\left[\begin{array}{c} 0\\ 0\\ \begin{array}{c} Tsin\theta \\ Tcos\theta \end{array} \end{array}\right]u_{k-1}+w_{k-1}$$

where $x_{k}={\left[x_{1},x_{2},{\dot{x}}_{1},{\dot{x}}_{2}\right]}^{T}$ is the state vector, with the first two states as the north and east position with the last two states as the north and east velocity of the target respectively. A sensor measures the position and velocity of the system as,

(42) $$z_{k}=Hx_{k}+v_{k}$$

with $H=\left[\begin{array}{ccc} 1 & 3 & \begin{array}{cc} 2 & 1 \end{array}\\ 3 & 1 & \begin{array}{cc} 0 & 1 \end{array}\\ \begin{array}{c} 2\\ 2 \end{array} & \begin{array}{c} 1\\ 2 \end{array} & \begin{array}{cc} \begin{array}{c} 3\\ 0 \end{array} & \begin{array}{c} 3\\ 1 \end{array} \end{array} \end{array}\right]$. $w_{k}$ and $v_{k}$ are the Gaussian process and measurement noise, respectively. Suppose that we have additional information that the vehicle is moving on the road with a heading angle of θ from the east $x_{2}$, then we can write,

$$\tan\theta =\frac{x_{1}}{x_{2}}\Rightarrow x_{1}-x_{2}\tan\theta =0$$

$$\tan\theta =\frac{{\dot{x}}_{1}}{{\dot{x}}_{2}}\Rightarrow {\dot{x}}_{1}-{\dot{x}}_{2}\tan\theta =0$$

Due to the heading of the vehicle, the states are dependent on each other, thus providing us additional constraints,

(43) $$\left[\begin{array}{c} \begin{array}{ccc} 1 & -\tan\theta & \begin{array}{cc} 0 & 0 \end{array} \end{array}\\ \begin{array}{ccc} 0 & 0 & \begin{array}{cc} 1 & -\tan\theta \end{array} \end{array} \end{array}\right]x_{k}=\left[\begin{array}{c} 0\\ 0 \end{array}\right]$$

Based on the constraint matrix $M={\left[\begin{array}{cc} I_{4} & H \end{array}\right]}^{T}$ between the state prediction and sensor measurement and linear constraints (43) among the state variables, the unified constraint matrix $M_{c}$ can be obtained as,

(44) $$M_{c}={\left[\begin{array}{cccc} \begin{array}{c} \tan\theta \\ 0 \end{array} & \begin{array}{c} 1\\ 0 \end{array} & \begin{array}{c} 0\\ \tan\theta \end{array} & \begin{array}{cccc} \begin{array}{c} 0\\ 1 \end{array} & \begin{array}{c} 3+\tan\theta \\ 1+2\tan\theta \end{array} & \begin{array}{c} 1+3\tan\theta \\ 1 \end{array} & \begin{array}{cc} \begin{array}{c} 1+2\tan\theta \\ 3+3\tan\theta \end{array} & \begin{array}{c} 2+2\tan\theta \\ 1 \end{array} \end{array} \end{array} \end{array}\right]}^{T}$$

The covariance of the process and measurement noise are assumed to be,

$$Q=\left[\begin{array}{ccc} 100 & 20 & \begin{array}{cc} 0 & 0 \end{array}\\ 20 & 100 & \begin{array}{cc} 0 & 0 \end{array}\\ \begin{array}{c} 0\\ 0 \end{array} & \begin{array}{c} 0\\ 0 \end{array} & \begin{array}{cc} \begin{array}{c} 30\\ 10 \end{array} & \begin{array}{c} 10\\ 30 \end{array} \end{array} \end{array}\right],\;R=\left[\begin{array}{ccc} 500 & 10 & \begin{array}{cc} 20 & 30 \end{array}\\ 10 & 500 & \begin{array}{cc} 15 & 10 \end{array}\\ \begin{array}{c} 20\\ 30 \end{array} & \begin{array}{c} 15\\ 10 \end{array} & \begin{array}{cc} \begin{array}{c} 100\\ 20 \end{array} & \begin{array}{c} 20\\ 100 \end{array} \end{array} \end{array}\right]$$

The target is set to an initial state of $\left[\begin{array}{ccc} 0 & 0 & \begin{array}{cc} 15\tan\theta & 15 \end{array} \end{array}\right]$. The sampling time *T* is set to 1 s, heading angle θ=π/3 and control input $u_{k}=1$ if ${\dot{x}}_{1}\;or\;{\dot{x}}_{2}<30$ otherwise it is changed to −1 until ${\dot{x}}_{1}\;or\;{\dot{x}}_{2}<5.$ The process and measurement noise are assumed to be correlated with covariance,

$$P_{QR}=\rho F_{1}F_{2}^{T}$$

where $F_{1}$ and $F_{2}$ are the cholesky decomposition of Q and R respectively. ρ is the correlation coefficient and assumed as 0.4 in the simulation.

**Figure 10:**
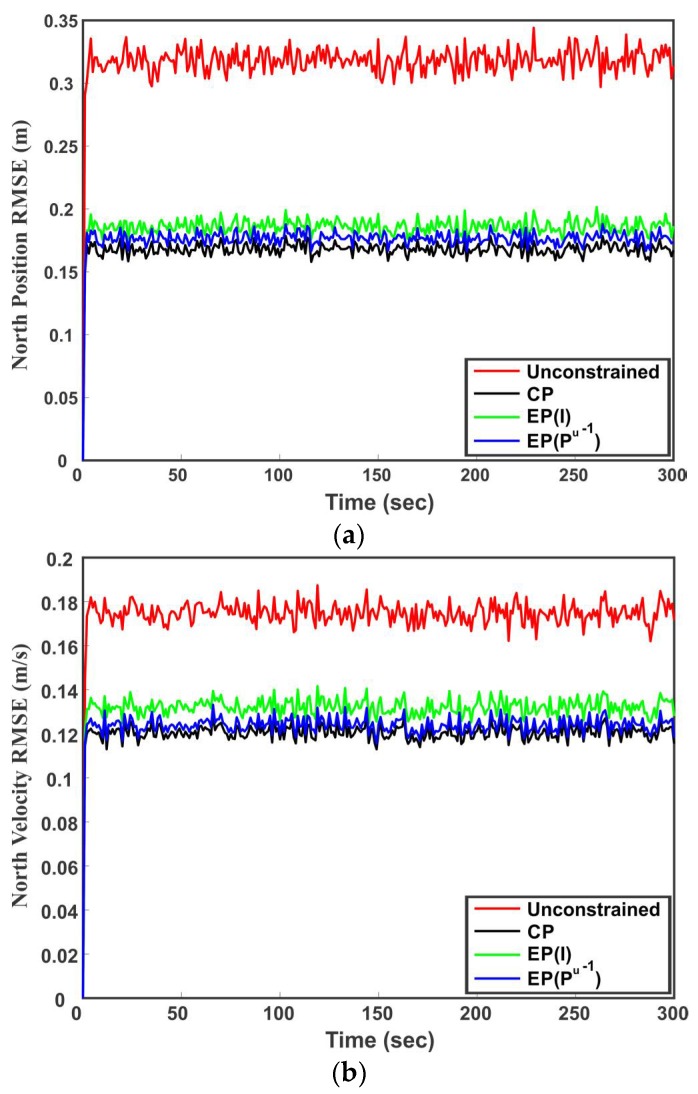
Simulation results for the constrained and unconstrained dynamic system. The covariance projection (CP) method is compared with the unconstrained state estimate and estimate projection (EP) method. (**a**) RMSE of northerly position of vehicle over 1000 runs; (**b**) RMSE of northerly velocity of vehicle over 1000 runs.

**Figure 11:**
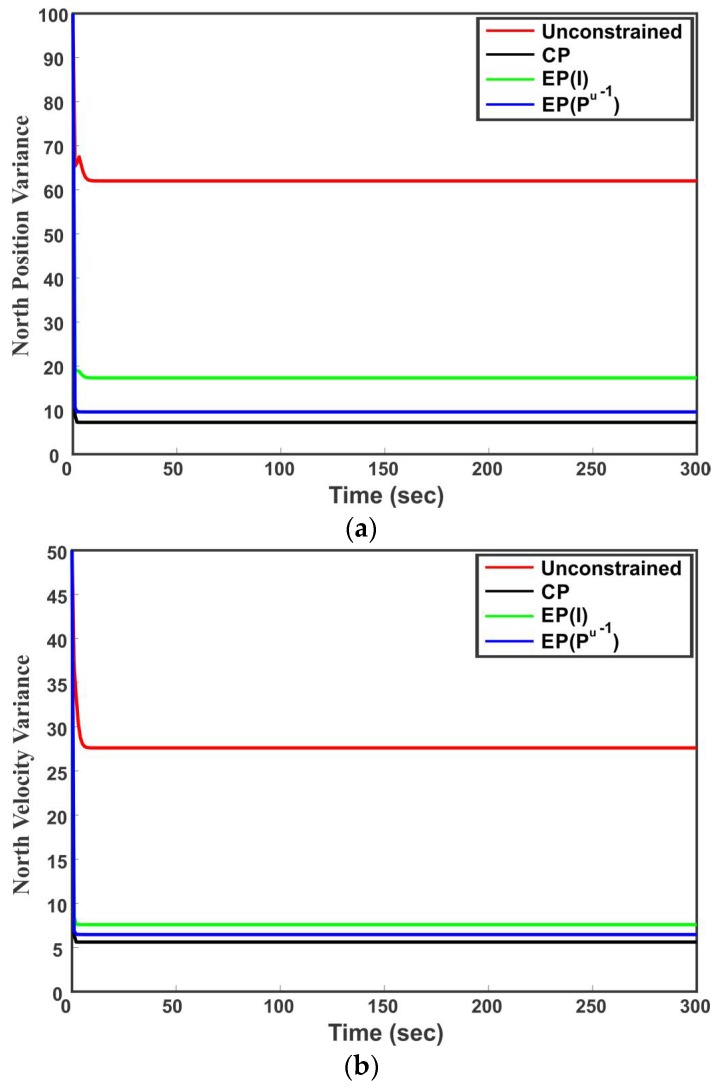
Comparative results of different methods in terms of states variance. (**a**) Variance of the northerly position of vehicle; (**b**) Variance of the northerly velocity of vehicle.

Starting from the initial value, in each time step (41) is used to predict the states of the vehicle. The states are then updated with the sensor measurements (42). Using $M_{c}$ from (44) in (34) and (35), the proposed method directly satisfies the constraint between the perdition and measurement as well as the linear constraints among states due to the heading of the vehicle. On the other hand, the EP method first employs a Kalman filter to obtain the unconstrained state estimates and then project the unconstrained state estimates on the linear constraints subspace to satisfy the constraints among states. The performance of the proposed method is compared with the unconstrained estimate and estimate provided by the EP method in terms of RMSE. Table 3 summarizes the average RMSE of the individual states for 1000 Monte Carlo runs. It can be observed from Table 3 that the RMSE of the CP method is lower than the other estimators for all states. For the EP method, the use of $P^{u^{-1}}$ as a weighting parameter provides better results than using I. The comparative results of different methods can be also seen in Figure 10a,b, where Figure 10a is the RMSE of the northerly position and Figure 10b is the RMSE of northerly velocity. It can be seen that the proposed method performs better as compared to the unconstrained state estimation and EP method. Figure 11a,b show the variance of northerly position and northerly velocity of the vehicle, respectively. It is proven that the EP method with weighting parameter $P^{u^{-1}}$ has the smallest covariance [24]. This can also be observed from Figure 11a,b where the EP method with weight $P^{u^{-1}}$ provides smaller variance than the unconstrained estimate and EP method by identitying matrix I as the weighting parameter. However, the CP method results in even smaller state variance than all the competing methods as seen in Figure 11a,b.

## 7. Conclusions

In this paper, we propose a general approach to data fusion under arbitrary correlations and linear constraints as well as data inconsistency. The proposed method provides an unbiased and optimal solution in the sense of MMSE for fusing data from multiple sources. The method improves the fusion accuracy by automatically detecting and removing inconsistent estimates from the fusion pool by a statistical confidence measure. Moreover, it is shown that by considering the cross-correlation among local estimates, the proposed method avoids the deterioration of the fusion accuracy due to Type I and Type II errors. Without any additional manipulation, the proposed method completely satisfies any linear constraints among state variables. The method improves the accuracy of constrained state estimation by satisfying the linear constraints among state variables and provides better results than the unconstrained state estimation and constrained state estimation using the estimate projection method.

Future work includes the extension of the proposed method for state estimation of non-linear dynamic systems. Another avenue is examining the proposed method for incorporating non-linear constraints among state variables.

## Figures and Tables

**Table 1:** The accuracy comparison in terms of trace of matrices.

$traceP_{1}$	$traceP_{2}$	$traceP_{3}$	$traceP_{4}$	$trace\tilde{P}$
4.3271	2.9109	3.9656	3.4321	1.2674

**Table 2:** Average RMSE for 1000 Monte Carlo Runs.

Average RMSE	CP	CP WO-d	CP WO-dC
Position (m)	101.1605	93.0824	89.6491
Velocity (m/s)	43.0339	36.0899	31.2033

**Table 3:** Average RMSE for 1000 Monte Carlo Runs.

Methods	Average RMSE			
	$x_{1}$ (m)	$x_{2}$ (m)	${\dot{x}}_{1}$ (m/s)	${\dot{x}}_{2}$ (m/s)
Unconstrained	95.479	87.496	52.413	49.972
CP	50.39	29.093	36.624	21.145
EP (I)	55.823	32.229	39.648	22.891
EP ($P^{u^{-1}}$)	53.023	30.613	37.186	21.469

## Appendix A

The fused mean estimate and covariance of the covariance projection (CP) method are given as,

(A1) $$\tilde{x}=W^{-1}P_{r}W\hat{x}$$

(A2) $$\tilde{P}=W^{-1}P_{r}WPW^{T}{P_{r}}^{T}W^{-T}$$

Putting $W=D^{-1/2}E^{T},$ $P_{r}=M^{W}{\left(M^{W^{T}}M^{W}\right)}^{-1}M^{W^{T}}$ and $M^{W}=WM$ in (A2), we get,

$$\tilde{P}=W^{-1}\left(WM{\left(M^{T}W^{T}WM\right)}^{-1}M^{T}W^{T}\right)\times {\left(WM{\left(M^{T}W^{T}WM\right)}^{-1}M^{T}W^{T}\right)}^{T}W^{-T}$$

Let $\alpha =M^{T}W^{T}WM$, then,

$$\tilde{P}=W^{-1}WM\alpha^{-1}M^{T}W^{T}WM\alpha^{-T}M^{T}W^{T}W^{-T}$$

(A3) $$\tilde{P}=M\alpha^{-T}M^{T}$$

Putting the value of α in (A3) and simplifying, we get,

(A4) $$\tilde{P}=M{\left(M^{T}ED^{-1}E^{T}M\right)}^{-1}M^{T}$$

By eigenvalue decomposition, we know that,

$$P=EDE^{T}\Rightarrow P^{-1}=ED^{-1}E^{T}$$

So,

(A5) $$\tilde{P}=M{\left(M^{T}P^{-1}M\right)}^{-1}M^{T}$$

Similarly, using definitions of various components in CP fused mean (A1), we have,

$$\tilde{x}=W^{-1}\left(WM{\left(M^{T}W^{T}WM\right)}^{-1}M^{T}W^{T}\right)W\hat{x}$$

(A6) $$\tilde{x}=M{\left(M^{T}W^{T}WM\right)}^{-1}M^{T}W^{T}W\hat{x}$$

Since $W^{T}W=P^{-1}$, (A6) can be simplified as,

(A7) $$\tilde{x}=M{\left(M^{T}P^{-1}M\right)}^{-1}M^{T}P^{-1}\hat{x}$$

## Appendix B

The weighted distance from the joint mean of two data sources to the point on the manifold can be calculated as,

$$d=\left[\begin{array}{cc} {\left({\hat{x}}_{1}-\tilde{x}\right)}^{T} & {\left({\hat{x}}_{2}-\tilde{x}\right)}^{T} \end{array}\right]{\left[\begin{array}{cc} P_{1} & 0\\ 0 & P_{2} \end{array}\right]}^{-1}\left[\begin{array}{c} \left({\hat{x}}_{1}-\tilde{x}\right)\\ \left({\hat{x}}_{2}-\tilde{x}\right) \end{array}\right]$$

(A8) $$d={\left({\hat{x}}_{1}-\tilde{x}\right)}^{T}P_{1}^{-1}\left({\hat{x}}_{1}-\tilde{x}\right)+{\left({\hat{x}}_{2}-\tilde{x}\right)}^{T}P_{2}^{-1}\left({\hat{x}}_{2}-\tilde{x}\right)$$

$${\hat{x}}_{1}-\tilde{x}={\hat{x}}_{1}-P_{2}{\left(P_{1}+P_{2}\right)}^{-1}{\hat{x}}_{1}+P_{1}{\left(P_{1}+P_{2}\right)}^{-1}{\hat{x}}_{2}$$

$${\hat{x}}_{1}-\tilde{x}=\left[I-P_{2}{\left(P_{1}+P_{2}\right)}^{-1}\right]{\hat{x}}_{1}-\left[P_{1}{\left(P_{1}+P_{2}\right)}^{-1}\right]{\hat{x}}_{2}$$

Since $P_{1}{\left(P_{1}+P_{2}\right)}^{-1}+P_{2}{\left(P_{1}+P_{2}\right)}^{-1}=I$

(A9) $${\hat{x}}_{1}-\tilde{x}=P_{1}{\left(P_{1}+P_{2}\right)}^{-1}\left[{\hat{x}}_{1}-{\hat{x}}_{2}\right]$$

Similarly,

$${\hat{x}}_{2}-\tilde{x}=\left[I-P_{1}{\left(P_{1}+P_{2}\right)}^{-1}\right]{\hat{x}}_{2}-\left[P_{2}{\left(P_{1}+P_{2}\right)}^{-1}\right]{\hat{x}}_{1}$$

$${\hat{x}}_{2}-\tilde{x}=P_{2}{\left(P_{1}+P_{2}\right)}^{-1}\left[{\hat{x}}_{2}-{\hat{x}}_{1}\right]$$

(A10) $${\hat{x}}_{2}-\tilde{x}=-P_{2}{\left(P_{1}+P_{2}\right)}^{-1}\left[{\hat{x}}_{1}-{\hat{x}}_{2}\right]$$

Putting (A9) and (A10) in (A8) we get,

$$d={\left(P_{1}{\left(P_{1}+P_{2}\right)}^{-1}\left[{\hat{x}}_{1}-{\hat{x}}_{2}\right]\right)}^{T}P_{1}^{-1}\left(P_{1}{\left(P_{1}+P_{2}\right)}^{-1}\left[{\hat{x}}_{1}-{\hat{x}}_{2}\right]\right)\;\;+{\left(-P_{2}{\left(P_{1}+P_{2}\right)}^{-1}\left[{\hat{x}}_{1}-{\hat{x}}_{2}\right]\right)}^{T}P_{2}^{-1}\left(-P_{2}{\left(P_{1}+P_{2}\right)}^{-1}\left[{\hat{x}}_{1}-{\hat{x}}_{2}\right]\right)$$

$$d=\left({\left[{\hat{x}}_{1}-{\hat{x}}_{2}\right]}^{T}{\left(P_{1}+P_{2}\right)}^{-1}P_{1}\right)P_{1}^{-1}\left(P_{1}{\left(P_{1}+P_{2}\right)}^{-1}\left[{\hat{x}}_{1}-{\hat{x}}_{2}\right]\right)\;\;+\left({\left[{\hat{x}}_{1}-{\hat{x}}_{2}\right]}^{T}{\left(P_{1}+P_{2}\right)}^{-1}P_{2}\right)P_{2}^{-1}\left(P_{2}{\left(P_{1}+P_{2}\right)}^{-1}\left[{\hat{x}}_{1}-{\hat{x}}_{2}\right]\right)$$

$$d={\left[{\hat{x}}_{1}-{\hat{x}}_{2}\right]}^{T}\left[{\left(P_{1}+P_{2}\right)}^{-1}\left(P_{1}+P_{2}\right){\left(P_{1}+P_{2}\right)}^{-1}\right]\left[{\hat{x}}_{1}-{\hat{x}}_{2}\right]$$

(A11) $$d={\left[{\hat{x}}_{1}-{\hat{x}}_{2}\right]}^{T}{\left(P_{1}+P_{2}\right)}^{-1}\left[{\hat{x}}_{1}-{\hat{x}}_{2}\right]$$

## References

[1] Liggins M., Hall D., Llinas J. (2017). Handbook of Multisensor Data Fusion: Theory and Practice.

[2] Hall D., Chong C., Llinas J., Liggins M. (2012). Distributed Data Fusion for Network-Centric Operations.

[3] Kalman R. (1960). A new approach to linear filtering and prediction problems. J. Basic Eng..

[4] Bar-Shalom Y. (1981). On the track-to-track correlation problem. IEEE Trans. Automat. Contr..

[5] Bakr M.A., Lee S. (2017). Distributed Multisensor Data Fusion under Unknown Correlation and Data Inconsistency. Sensors.

[6] Maybeck P. (1982). Stochastic Models, Estimation, and Control.

[7] Bar-Shalom Y., Campo L. (1986). The effect of the common process noise on the two-sensor fused-track covariance. IEEE Trans. Aerosp..

[8] Chang K.C., Saha R.K., Bar-Shalom Y. (1997). On optimal track-to-track fusion. IEEE Trans. Aerosp. Electron. Syst..

[9] Shin V., Lee Y., Choi T. (2006). Generalized Millman’s formula and its application for estimation problems. Signal Process..

[10] Sun S., Deng Z. (2004). Multi-sensor optimal information fusion Kalman filter. Automatica.

[11] Khaleghi B., Khamis A., Karray F., Razavi S. (2013). Multisensor data fusion: A review of the state-of-the-art. Inf. Fusion.

[12] Abdulhafiz W., Khamis A. (2013). Handling data uncertainty and inconsistency using multisensor data fusion. Adv. Artif. Intell..

[13] Durovic Z., Kovacevic B. (1995). QQ-plot approach to robust Kalman filtering. Int. J. Control.

[14] Wellington S., Atkinson J., Sion R. Sensor validation and fusion using the Nadaraya-Watson statistical estimator. Proceedings of the Fifth International Conference on Information Fusion.

[15] Hage J.A., Najjar M.E., Pomorski D. (2017). Multi-sensor fusion approach with fault detection and exclusion based on the Kullback–Leibler Divergence: Application on collaborative multi-robot system. Inf. Fusion.

[16] Del Gobbo D., Napolitano M., Famouri P., Innocenti M. (2001). Experimental application of extended Kalman filtering for sensor validation. IEEE Trans. Control Syst. Technol..

[17] Brumback B., Srinath M. (1987). A fault-tolerant multisensor navigation system design. IEEE Trans. Aerosp. Electron. Syst..

[18] Kumar M., Garg D., Zachery R. (2007). A method for judicious fusion of inconsistent multiple sensor data. IEEE Sens. J..

[19] Kumar M., Garg D., Zachery R. A generalized approach for inconsistency detection in data fusion from multiple sensors. Proceedings of the 2006 American Control Conference.

[20] Uhlmann J. (2003). Covariance consistency methods for fault-tolerant distributed data fusion. Inf. Fusion.

[21] Kirubarajan T., Bar-Shalom Y., Pattipati K., Kadar I. (2000). Ground target tracking with variable structure IMM estimator. IEEE Trans. Aerosp. Electron. Syst..

[22] Bernstein D.S., Hyland D.C. (1993). Compartmental Modeling and Second-Moment Analysis of State Space Systems. SIAM J. Matrix Anal. Appl..

[23] Simon D. (2010). Kalman filtering with state constraints: a survey of linear and nonlinear algorithms. IET Control Theory Appl..

[24] Simon D., Chia T. (2002). Kalman filtering with state equality constraints. IEEE Trans. Aerosp. Electron. Syst..

[25] Wen W., Durrant-Whyte H. Model-based multi-sensor data fusion. Proceedings of the 1992 IEEE International Conference on Robotics Automation.

[26] Tahk M., Speyer J. (1990). Target tracking problems subject to kinematic constraints. IEEE Trans. Automat. Contr..

[27] Porrill J. (1988). Optimal Combination and Constraints for Geometrical Sensor Data. Int. J. Robot. Res..

[28] De Geeter J., Van Brussel H., De Schutter J., Decreton M. (1997). A smoothly constrained Kalman filter. IEEE Trans. Pattern Anal. Mach. Intell..

[29] Hewett R.J., Heath M.T., Butala M.D., Kamalabadi F. (2010). A Robust Null Space Method for Linear Equality Constrained State Estimation. IEEE Trans. Signal Process..

[30] Bakr M.A., Lee S. A general framework for data fusion and outlier removal in distributed sensor networks. Proceedings of the 2017 IEEE International Conference on Multisensor Fusion and Integration for Intelligent Systems (MFI).

[31] Simon D. (2006). Optimal State Estimation: Kalman, H [Infinity] and Nonlinear Approaches.

[32] Jiang L. (2011). Sensor Fault Detection and Isolation Using System Dynamics Identification Techniques. Ph.D. Thesis.

[33] Bar-Shalom Y., Willett P., Tian X. (2011). Tracking and Data Fusion.

[34] Walpole R., Myers R., Myers S., Ye K. (1993). Probability and Statistics for Engineers and Scientists.

[35] Fishman G.S. (1996). Monte Carlo.

[36] Rillo G., Morales M.A., Ceperley D.M., Pierleoni C. (2018). Coupled electron-ion Monte Carlo simulation of hydrogen molecular crystals. J. Chem. Phys..

[37] Baudry G., Macharis C., Vallée T. (2017). Range-based Multi-Actor Multi-Criteria Analysis: A combined method of Multi-Actor Multi-Criteria Analysis and Monte Carlo simulation to support participatory decision making under uncertainty. Eur. J. Oper. Res..

[38] Ma Y., Chen X., Biegler L.T. (2018). Monte-Carlo-simulation-based optimization for copolymerization processes with embedded chemical composition distribution. Comput. Chem. Eng..

[39] Vîlcu A.-D., Vîlcu G.-E. (2018). An algorithm to estimate the vertices of a tetrahedron from uniform random points inside. Ann. Mat. Pura Appl..

[40] Hua X., Cheng Y., Wang H., Qin Y. (2018). Robust Covariance Estimators Based on Information Divergences and Riemannian Manifold. Entropy.

[41] Ljung L., Prochazka A., Kinsbury N., Payner P.J.W., Uhlir L. (1998). System Identification. Signal Analysis and Prediction.

